# The Remarkable Structural Diversity Achieved in *ent*-Kaurane Diterpenes by Fungal Biotransformations 

**DOI:** 10.3390/molecules19021856

**Published:** 2014-02-10

**Authors:** Jacqueline A. Takahashi, Dhionne C. Gomes, Fernanda H. Lyra, Gabriel F. dos Santos, Leonardo R. Martins

**Affiliations:** 1 Departamento de Química, Universidade Federal de Minas Gerais (UFMG), Av. Antonio Carlos, 6627, CEP 31270-901, Belo Horizonte, M.G., Brazil; E-Mails: fernandahl@yahoo.com.br (F.H.L.); gfsantos@ymail.com (G.F.S.); 2 Faculdade de Farmácia, Universidade Federal de Minas Gerais (UFMG), Av. Antonio Carlos, 6627, CEP 31270-901, Belo Horizonte, M.G., Brazil; E-Mail: dhionnegomes@hotmail.com; 3 Faculdade de Ciências Exatas e Tecnologia (FACET), Universidade Federal da Grande Dourados (UFGD), Rodovia Dourados-Itahum, km 12, CEP 79.804-970, Cx. Postal 533, Dourados, M.S., Brazil; E-Mail: leonardomartins@ufgd.edu.br

**Keywords:** biotransformation, kaurane diterpenes, filamentous fungi, hydroxylation, structure diversification

## Abstract

The use of biotransformations in organic chemistry is widespread, with highlights of interesting applications in the functionalization of natural products containing unactivated carbons, like the kaurane diterpenes. A number of compounds with kaurane skeletons can be isolated in large amounts from several plant species and a myriad of biological activities has been related to these compounds. Studies on structure *versus* activity have showed that, in most cases, in kaurane diterpenes, activity increases with the increase of functionalization. Since naturally occurring kaurane diterpenes usually have limited functional groups to be used as targets for semi-synthetic modifications, production of more polar derivatives from kaurane diterpenes have been achieved mostly through the use of fungal biotransformations. In this review, selected examples the wonderful chemical diversity produced by fungi in kaurane diterpenes is presented. This diversity includes mainly hydroxylation of nearly all carbon atoms of the kaurane molecule, many of them carried out stereoselectively, as well as ring rearrangements, among other chemical modifications. Sources of starting materials, general biotransformation protocols employed, fungi with most consistent regioselectivity towards kaurane skeleton, as well as biological activities associated with starting materials and products are also described.

## 1. Introduction

One of the major aims of natural products chemistry is the isolation of molecules with biological properties. With the development of novel strategies for natural products research, especially bio-monitored isolation and the use of preparative/hyphenated High Performance Liquid Chromatography (HPLC) there was a speed up in the discovery process, leading to a number of new molecules of diversified structures displaying an extensive range of bioactivities. At the same time, organic synthesis is producing novel molecules throughout total synthesis as well as a number of natural product derivatives for structure-activity studies. Biotransformations have an interesting role in a gap left between these two approaches, since they enable chemical modifications, mainly the introduction of hydroxyl groups, at inactivated carbons, which is a difficult task for semi-synthesis. The biotransformation approach has vast application in terpene molecules, a group of natural compounds possessing a myriad of biological activities, but however bearing only a few reactive sites for chemical modifications. Low polarity terpenes are usually available from natural sources in large amounts, making possible their prompt use in biotransformations. Biotransformation represents the second generation process of choice in the manufacture of small molecules with pharmaceutical applications [1].

The biotransformation of terpenes by fungi has applications in the most diverse areas, such as the pharmaceutical, food, cosmetics, and fragrance industries [2,3]. Nearly all classes of terpenes have been successfully functionalized by biotransformations. For instance, a monoterpene commonly targeted is limonene, due to its wide availability from cheap sources such as waste from the orange juice industry and its great potential as a substrate for the production of fragrances [4]. Functionalized derivatives of sesquiterpenes [5,6] and triterpenes [7] have also been obtained as fungal metabolites. Reports on the biotransformation of many different types of diterpenes, such as labdane [8], trachylobane [9,10], stemodane [11], aphidicolane [12], pimaradiene [13], abietane [14], taxane [15], atisane [16], and beyerane [17] are also very frequently found in the literature.

Kaurane diterpenes represent a target of interest for biotransformations because they constitute a class of substances rich in different biological activities [18]; some of them also act as intermediates in the biosynthesis of some fungal metabolites. The use of kaurane diterpenes in microbiological conversions is mainly directed to biosynthesis studies and to the production of derivatives with biological activities. In Table 1, an overview of some key biological activities related to kaurane diterpenes is presented.

**Table 1:** Biological activities associated with kaurane diterpenes.

Compound	Type	Plant or fungi (biotransformation) source	Biological activities	Reference
*ent*-18-acetoxykaur-16-ene	NP	*Annona squamosa*	anti-inﬂammatory and analgesic	[19]
*ent*-2α,16β,17-trihydroxykauran-19-oic acid and *ent-*3α,16β,17-trihydroxykauran-19-oic acid	NP	*Mikania hirsutissima*	immunomodulatory (human lymphocytes)	[20]
*ent-* 3β,15β,18-trihydroxykaur-16-ene	BP	*Mucor plumbeus* (fungus)	anti-allergic	[21]
*ent* 7β,16β,17-trihydroxykauran-6-one and *ent-*7α,16β,17-trihydroxykauran-6-one	NP	*Broussonetia papyrifera*	anti-tyrosinase	[22]
*ent*-13,16β,17-trihydroxykauran-19-oic acid	BP	*Mucor recurvatus* (fungus)	glucocorticoid agonists	[23]
*ent*-3β,15β,18-trihydroxykaur-16-ene *ent*-3-oxo-15β,18-dihydroxykaur-16-ene and *ent*-3β,15β-dihydroxykaur-16-ene	NP	*Suregada multiflora*	anti-allergic	[24]
*ent*-16βH,17-isobutyryloxykauran-19-oic acid and *ent*-16βH,17-acetoxy-18-isobutyryloxykauran-19-oic acid	NP	*Siegesbeckia glabrescens*	antidiabetic and antiobesity	[25]
*ent*-2α-hydroxy,16-oxo-17-norkauran-19-oic acid	BP	*Fusarium proliferatum* (fungus)	allelopathic	[26]
*ent*-7α,11β-dihydroxykaur-16-en-19-oic acid and *ent*-1β,7α-dihydroxykaur-16-en-19-oic acid	BP	*Aspergillus niger* (fungus)	spasmolytic	[27]
*ent*-16β,19-dihydroxykaurane and *ent*-16β,17,19-trihydroxykaurane	BP	*Cephalosporium aphidicola* (fungus)	allelopathic	[28]
*ent*-kaur-16-en-19-oic acid (kaurenoic acid)	NP	*Aspilia foliacea*	antimicrobial	[29]
	NP	*Mikania obtusata, Xylopia frutescens, X. sericea* and *Wedelia paludosa*	trypanocidal	[30,31]
	NP	*Melantheria albinervia*	larvicidal	[32]
	NP	*Annona glabra*	antimicrobial, antifungal, antihelmintic and sporicidal	[33]
	NP	*W. paludosa*	antinociceptive	[34]
	NP	*Copaifera langsdorfﬁi*	cytotoxic and genotoxic	[35,36]
	NP	*W. paludosa*	anti-inflammatory	[37]
	NP	*Laetia thamnia*	anti-Parkinsonism	[38]

NP: Natural Product isolated from plants; BP: Biotransformation Product obtained using fungi.

**Figure 1:**
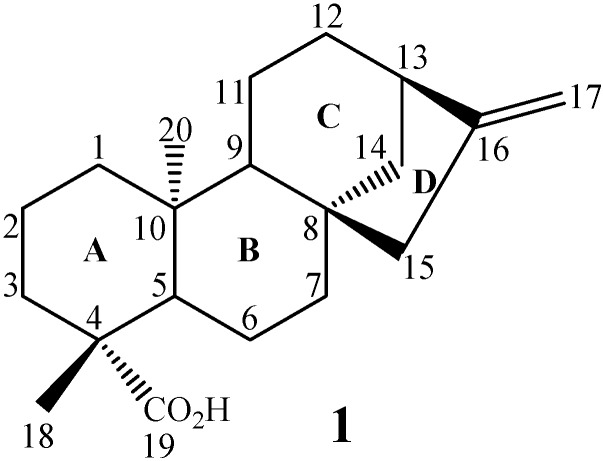
Chemical structure of kaurenoic acid.

Among these compounds, *ent-*kaur-16-en-19-oic acid (kaurenoic acid, **1**, Figure 1) is one of the most studied compounds, as it has several and varied biological activities, such as antimicrobial [29], inhibition of HIV replication [39], anti-inflammatory [40], trypanocidal [41] and cytotoxic properties [42], among others.

**Figure 2:**
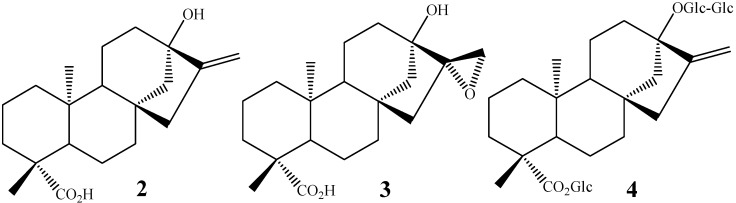
Chemical structures of steviol (**2**), steviol 16α,17-epoxide (**3**) and stevioside (**4**).

Other kaurane diterpenes like steviol (**2**), steviol 16α,17-epoxide (**3**) and stevioside (**4**) (Figure 2), showed anti-inflammatory activity when screened as glucocorticoid agonists [43].

Hydroxylated kaurane diterpenes are of special interest, as oxygenated derivatives generally have higher levels of biological activity than their non-polar precursors. For instance, Ohkoshi *et al.* reported significant immunomodulatory activity towards human lymphocytes for kaurenoic acid derivatives hydroxylated in carbons 2, 3, 16 and 17 [20]. Kaurane compounds bearing a 6β-OH group that establishes a hydrogen bond with a C-15 carbonyl group have been associated to antitumor activity as well as antibacterial activity against Gram-positive bacteria [44].

Evaluation of the relationship between structure and antimicrobial activity of some diterpenes suggested that the presence of a substituted decalin skeleton (hydrophobic portion), and a hydrophilic region bearing a donor-group able to interact with hydrogen-bond acceptor groups on the membrane were necessary for their insertion in a phospholipid bilayer model. This feature enables the diterpenes to cross the bacterial cell membrane causing cell damage [45].

**Figure 3:**
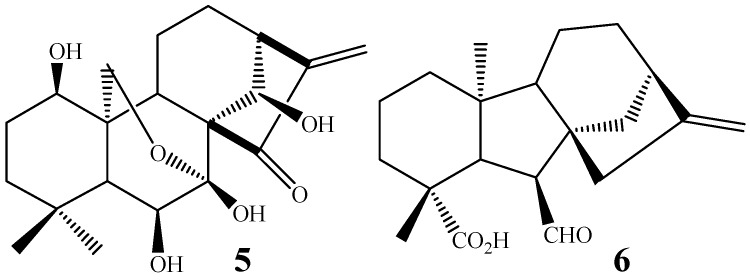
Chemical structures of compounds **5** and **6**.

Other studies reported that increase in the antitumor activity in kaurane diterpenes is related to the presence of hydroxyl groups at C-7 and/or C-14 [46,47]. Polyhydroxylated kaurane diterpenes like oridonin (**5**, Figure 3) present anti-tumor activity against P 388 lymphocytic leukemia in mice, and increases in the degree of functionalization have been associated with higher antitumor activity [48].

The hydroxylation of C-11 in kaurane diterpenes is frequently accomplished by fungal biotransformation. These molecules hold some similarities to the steroidal nucleus. The presence of a hydroxyl group at C-11 in the steroidal nucleus is a structural requirement for carbohydrate-regulating hormonal activity of the adrenal steroids [49]. Cortisol and aldosterone are good examples of steroidal hormones hydroxylated at C-11, and hydroxylation of these molecules is catalyzed by 11β-hydroxylase and aldosterone synthase enzymes that belong to the cytochrome P 450 complex [50].

## 2. Biotransformations of Kaurane Diterpenes by Fungi from *Gibberella fujikuroi* Complex

Many biotransformations of kaurane diterpenes described in the literature have been carried out by *G. fujikuroi.* The advancement of genetic sequencing techniques allowed gathering at least 50 different species of fungi of *Fusarium* genus including *F. proliferatum, F. acutatum* and *F. fujikuroi* within a group called *G. fujikuroi* complex [51]. This group of fungi has the enzymatic system conducive to carry on the same type of biotransformation, and is reported as producers of diterpenes [52]. The *G. fujikuroi* complex was gathered due to the need to reclassify different species of fungi from *Fusarium* genus, which were classified according to the same taxonomy, as they possessed identical morphological features.

The kaurane diterpenes are produced through the mevalonate pathway. The first metabolite of this via contains a tetracyclic skeleton, produced from geranylgeranyl pyrophosphate through the action of the enzyme kaurene synthetase. Post-kaurene modifications are related to the cytochrome P-450, for example, oxygenases [52] and monooxygenases [53]. Kaurane diterpenes, like kaurenoic acid, are natural intermediates in the *in vivo* biosynthesis of gibberellins. Therefore, some kaurane diterpenes, under the action of fungi from *G. fujikuroi* complex, are hydroxylated in C-7, following a rearrangement with ring B contraction, into a five-membered ring, due to extrusion of C-7, providing gibberellins [54]. The gibberellins are tetracyclic diterpenes present in low concentrations in plants, where they regulate different stages of growth and development. An example is the production of *ent-*7-oxo-gibb-16-ene (**6**) (Figure 3) by the biotransformation of a kaurane precursor by *G. fujikuroi* [55]. The production of gibberellins from the biotransformation of kaurane diterpenes is of great importance and high commercial value, mainly because gibberellins natural production occurs in very small amounts [56,57].

**Figure 4:**
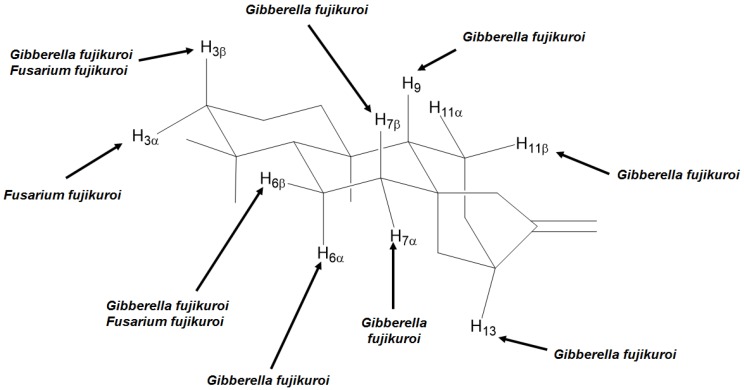
Positions and orientations of hydroxyl groups added to kaurane diterpenes by biotransformations using fungi from *G. fujikuroi* complex.

Fungi from *G. fujikuroi* complex were used as biocatalysts not only for producing gibberellins, but also for the hydroxylation of different positions, as shown in Figure 4.

Since kaurane diterpenes are not xenobiotic to fungi from the *G. fujikuroi* complex, these biotransformations are classified as biosynthetically directed biotransformations. The use of inhibitors of the natural biosynthesis is very common in this kind of experiments to avoid production of natural metabolites that otherwise may be confused with biotransformation products. Concerning to diterpenes, biotransformations using *G. fujikuroi* is usually accompanied by the use of biosynthesis inhibitors like AMO 1618 (2-isopropyl-4-dimethylamino-5-methylphenyl-1-piperidinecarboxylate methyl chloride). This inhibitor acts inhibiting *ent-*kaurene biosynthesis however does not affect post-kaurene metabolism [58].

## 3. Biotransformation Scope

Biotransformation experiments are mostly performed in aqueous environments in order to achieve the optimum growth of the micro-organism. This feature makes biotransformations green processes, however it may also result in low yields for non-optimized processes. In typical fungal biotransformation experiments, the substrates are usually added to the culture medium as dilute solutions since high amounts of substrate can lead to growth inhibition or even death of the micro-organisms. Even when feeding dilute solutions to the fungi, high amounts of unaltered substrate is usually recovered. Products yields reported for initial non-optimized biotransformation experiments are typically below 10%, although yields reaching 50% to 60% have also been related [59].

In most literature reports, it is not clear whether the product yield was calculated from the amount of substrate fed to the fungus or from the amount of substrate fed after adjusting for the amount of unconsumed starting material recovered from the experiments. It is a consensus that the aqueous environment and amount of substrate fed to the fermentation is an important issue in biotransformation yields [1]. Isolation of biotransformation products is sometimes only possible after derivatization, like esterification of carboxyl groups.

Two stage cultures seems to have been more frequently used [31] than direct inoculation of biotransformation media. In two stage cultures, the fungus is firstly grown in the liquid medium (stage 1) and then transferred to a vessel containing fresh sterile liquid medium (stage 2), where the biotransformation will take place after further mycelia development, followed by substrate addition. In a number of successful biotransformation experiments, incubation times varied from 6 [60] to 14 days [23,26,28], although smaller or longer incubation times can be found. Shorter incubation time seems to be very suitable for biosynthetically directed biotransformations [53,61].

**Scheme 1:**
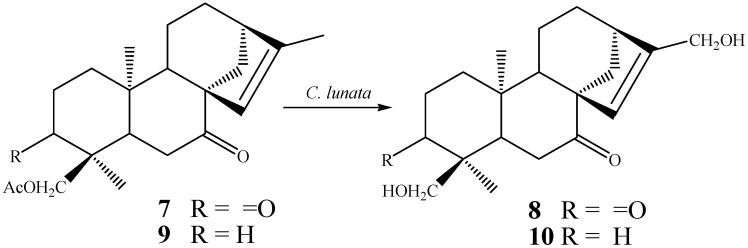
Biotransformation of *ent-*18-acetoxykaur-15-ene-3,7-dione (**7**) and *ent-*18-acetoxykaur-15-en-7-one (**9**) by the fungus *C. lunata*.

The literature shows that, in some cases, very short biotransformation periods can also result in products like the biotransformation of the diketone *ent-*18-acetoxykaur-15-ene-3,7-dione (**7**) by the fungus *Curvularia lunata* that furnished the product *ent-*17,18-dihydroxykaur-15-ene-3,7-dione (**8**) in very high yield (58%) after only 30 h [62]. In the same way, biotransformation of *ent-*18-acetoxykaur-15-en-7-one (**9**) with the fungus *C. lunata* furnished the product *ent-*17,18-dihydroxykaur-15-en-7-one (**10**) in 18% yield after only 48 h (Scheme 1) [62].

The nomenclature of kaurane diterpenes used in this review follows the guidelines described by Giles [63]. The prefix *ent*- before the name of the compounds means complete inversion at all configurations stated in relation to the fundamental parent structure.

## 4. Most Common Methylene Hydroxylation in Kaurane Diterpenes

The most common and also more attractive transformation carried out by fungi on kaurane structures is hydroxylation. This is of great value due to the possibility of achieving increased biological activities, as explained before. The following examples will illustrate that nearly all carbons of the kaurane skeleton have been activated with hydroxyl groups by the action of filamentous fungi.

**Scheme 2:**
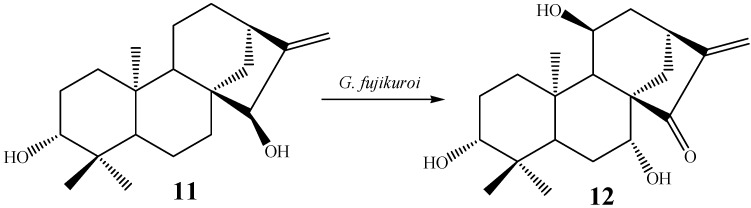
Biotransformation of *ent*-3β,15α-dihydroxy-kaur-16-ene (**11**) by *G. fujikuroi.*

Some of the most common hydroxylations of kaurane diterpenes seem to take place in carbons 7 and 11, either in the α- or β-positions. Carbons 3, 6, 9, and 13 have been hydroxylated less frequently by biotransformation, however these are also commonly described as hydroxylation sites. For instance, from a total of 65 randomly chosen papers about biotransformations of kaurane diterpenes (Table 2), 56 reported mostly hydroxylation of positions 7 (82.1%), 11 (41.1%), 6 (23.2%), 13 (14.3%), 3 and 9 (12.5% each), respectively. It is not uncommon to find examples in which both positions 7 and 11 were simultaneously hydroxylated. For instance, the biotransformation of *ent*-3β,15α-dihydroxy-kaur-16-ene (**11**) by the fungal species *G. fujijuroi* conducted by Fraga *et al.* [61], furnished *ent*-3β,7β,11α-trihydroxy-15-oxo-kaur-16-ene (**12**) (Scheme 2). Regarding the α- or β-orientation of the hydroxyl groups introduced in the molecules via fungal biotransformations, the β-orientation seems to be prefered, especially with regard to carbons 7 and 11. This may be result of the steric hindrance caused by carbons 18 and 20, both located on the α-face of kaurane compounds.

**Table 2:** Examples of fungi able to hydroxylate kaurane diterpenes, positions and stereochemistry of hydroxylations.

Fungal species	Positions and stereochemistry	References
*Absidia blakesleeana*	7β, 11α, 13	[64]
*Aspergillus niger*	3α, 7β, 11α	[23,27,65]
*Aspergillus ochraceus*	6β, 7α, 13	[66,67]
*Calonectria decora*	7α, 7β	[66,67]
*Cephalosporium aphidicola*	3α, 11β	[68,69,70]
*Cunninghamella bainieri*	9	[43]
*Cunninghamellha blakesleeana*	7β	[71]
*Fusarium fujikuroi*	3α, 3β, 6β, 7β	[60]
*Fusarium moniliforme*	11β	[72]
*Gibberella fujikuroi*	3β, 6α, 6β, 7α, 7β, 9, 11β, 13	[10,53,55,56,57,58,61,73,74,75,76,77,78,79,80,81,82,83,84,85,86,87,88,89,90,91,92,93,94,95,96,97,98,99,100]
*Mucor plumbeus*	6α, 7α, 9, 11β	[21,101,102]
*Mucor recurvatus*	7β, 11α, 11β	[23]
*Psylocybe cubensis*	11β	[103]
*Rhizopus nigricans*	3α, 7α, 7β, 13	[59,62,66,67]
*Rhizopus oligosporus*	7β, 9	[64]
*Rhizopus stolonifer*	7β, 9, 11β	[101,104,105]
*Verticillium lecanii*	7α, 7β, 11β	[31]

In addition to the positions and orientations mostly functionalized by biotransformation, another point of interest is the fungal species most employed in biotransformations of kaurane diterpenes. Table 2 presents the great variety of fungi employed in these reactions, the hydroxylation site and stereochemistry of the hydroxyl group introduced in the molecule. It can be noticed that some of these biotransformations consist of regioselective introduction of hydroxyl groups. Many other examples are available in the literature and some of them will also be discussed later in this review.

**Figure 5:**
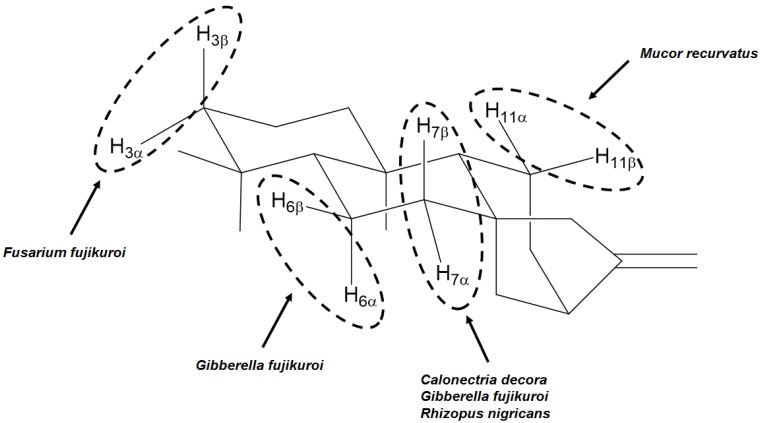
Examples of fungi able to hydroxylate both α and β positions of the same carbon of kaurane diterpenes.

It is interesting to observe that some species are able to hydroxylate the same carbon both on the α- and β-faces, such as carbon 3 by *F. fujikuroi*, or carbon 6 by *G. fujikuroi,* among other (Figure 5).

**Figure 6:**
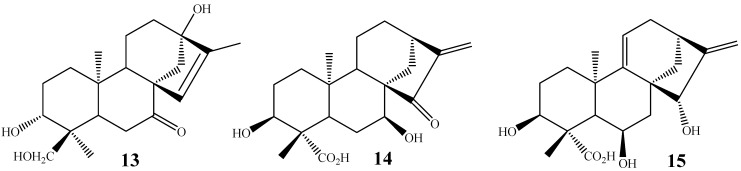
Chemical structures of hydroxylated derivatives **13**–**15**, obtained by the action of *R. nigricans* and *G. fujikuroi* on kaurane skeleton.

Some fungi have the ability of performing more than one hydroxylation in different positions of the starting materials. In this way, the biotransformation of *ent*-18-acetoxykaur-15-en-7-one by *R. nigricans* furnished the product **13** [59], while *ent*-15-oxo-kaur-16-ene led to the isolation of derivative **14** after biotransformation with *G. fujikuroi* [58]. In another example, *ent*-15β-hydroxy-kaur-9 (11),16-dien-19-oic acid [60] was biotransformed in the derivative **15** by *F. fujikuroi* (Figure 6).

**Figure 7:**
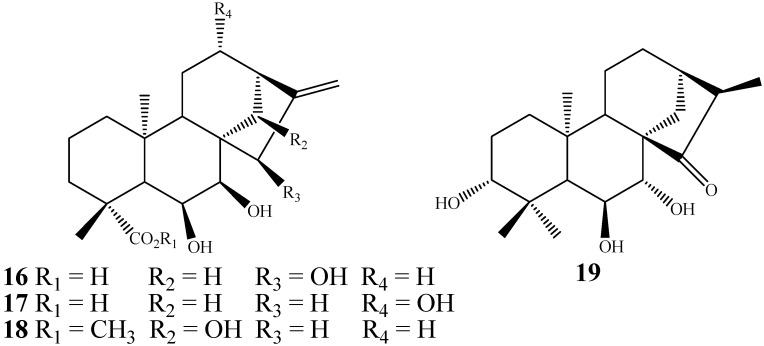
Chemical structures of the β,β and β,α kaurane vic-diols **16**–**19**.

*G. fujikuroi* can produce two vicinal hydroxylations on ring B to furnish *vic*- β,β- [75,86,95] and β,α-diols (Figure 7) [61].

**Figure 8:**
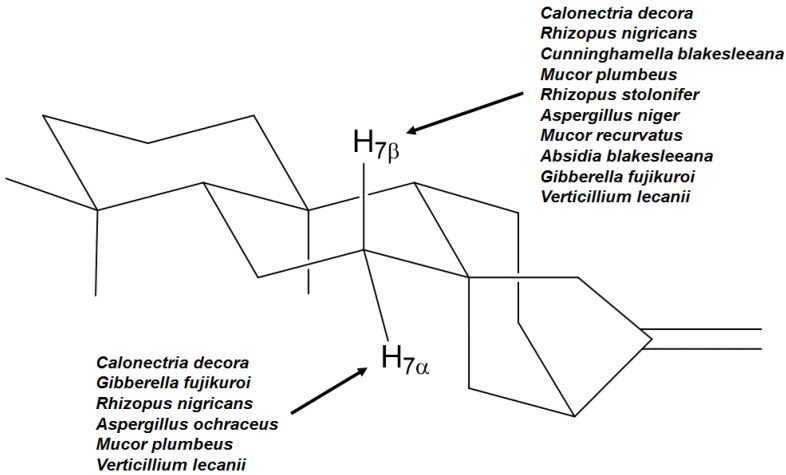
Examples of fungi able to carry on hydroxylation in carbon 7 of the kaurane skeleton.

The position C-7 is one of the most hydroxylated positions, regardless the fungal species used, as shown in Figure 8.

**Figure 9:**
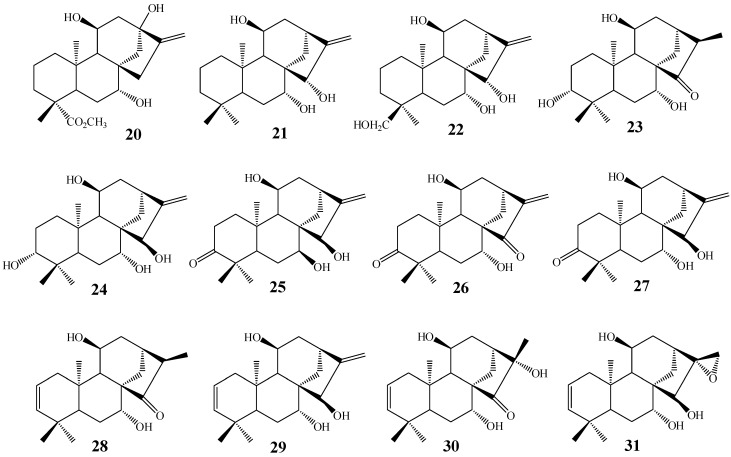
Chemical structures of the great structural diversity of biotransformation products hydroxylated in carbons 7 and 11 by the fungus *G. fujikuroi*.

Shigematsu *et al.* [84], and Fraga *et al.* [92,96,100] using *G. fujikuroi*, isolated a great diversity of C-7 and C-11 dihydroxylated products **20**–**31** (Figure 9).

**Figure 10:**
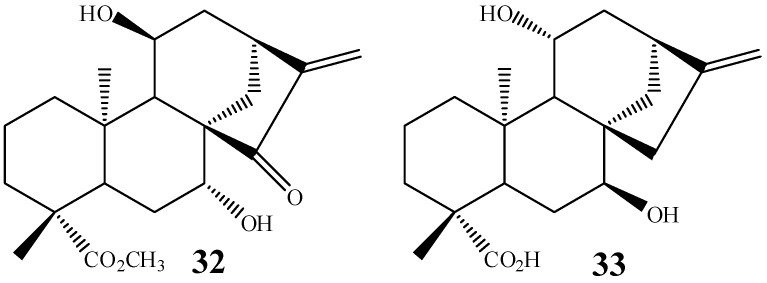
Chemical structures of products **32** and **33**.

Boaventura *et al.* [101], working with *M. plumbeus*, Marquina *et al.* [27] using *A. niger* and Taveepanich *et al.* [64] using the fungus *A. blakesleeana* also isolated 7,11 dihydroxylated products **32**, **33**, showing that this behavior is not unique to *G. fujikuroi* (Figure 10).

**Figure 11:**
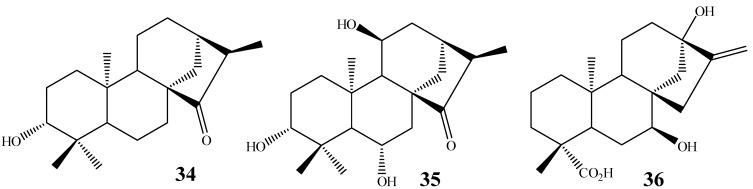
Chemical structures of starting material **34** and products hydroxylated in C-6/C-11 and C-7/C-13 by the fungi *G. fujikuroi* and *A. blakesleeana*.

Some variations are found, as, for example, Fraga *et al.* [61] reported the biotransformation of *ent*-3β-hydroxy-15-oxo-(16S)-kaurane (**34**) with the isolation of products hydroxylated in positions 6 and 11 (**35**) by biotransformation using *G. fujikuroi,* while Taveepanich *et al.* [64] also isolated a dihydroxylated product at positions 7 and 13 (**36**) of kaurenoic acid with the fungus *A. blakesleeana* (Figure 11).

Table 3 gives an interesting comparison of the yields obtained by two groups, working with the same fungi and closely related starting materials [66,67].

**Table 3:** Comparison on the yields obtained by Beilby *et al.* [66] and Ghisalberti *et al.* [67].

Substrate	Fungus	Positions hydroxylated	Yield [66] (%)	Yield [67] (%)
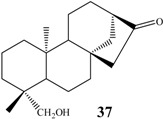 *ent*-19-hydroxy-16-oxo-17-nor-kaurane	*A. ochraceus*	7α	n.i.	20
	*C.decora*	1α 7α	10 10	10 10
	*R. nigricans*	1α 7α	20 20	20 20
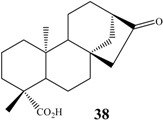 *ent*-16-oxo-17-nor-kauran-19-oic acid	*A. ochraceus*	13	5	5
	*C. decora*	1α 7α 7β	10 15 40	5 15 40
	*R. nigricans*	1α 7α 7β	30 30 5	30 30 5
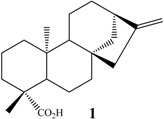 *ent*-kaur-16-en-19-oic acid	*A. ochraceus*	16α, 17	20	10
	*C. decora*	7α, 15α 15α 7α	30 5 5	30 5 5
	*R. nigricans*	16α, 17 7β	n.i. 25	10 25
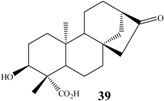 *ent*-3α-hydroxy-16-oxo-17-nor-kauran-19-oic acid	*A. ochraceus*	6β 7α	30 25	n.i. n.i.
	*C. decora*	7α	40	n.i.
	*R. nigricans*	1α 7α	25 35	n.i. n.i.

n.i.: not isolated.

## 5. Hydroxylations on Ring D

**Scheme 3:**
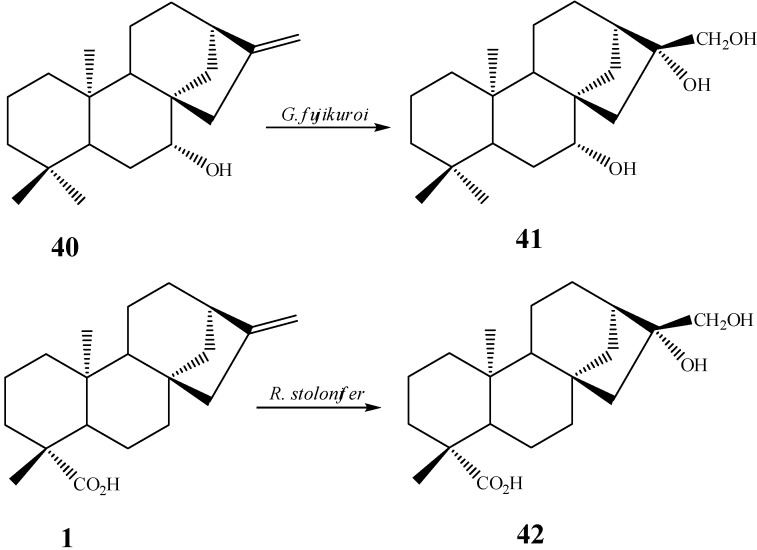
Hydroxylation of carbons 16 and 17 by fungi *G. fujikuroi* and *R. stolonifer.*

One of the most often reported structural changes accomplished by filamentous fungus on the ring D of kaurane diterpenes is the modification of the double bond between carbons 16 and 17, present in most of the compounds subjected to biotransformations. Incubation of *epi*-candol A (*ent*-7β-hydroxy-kaur-16-ene, **40**), with the fungus *G. fujikuroi* led to the formation of the diol *ent*-7β,16β,17-trihydroxy-kaur-16-ene (**41**) in 0.45% yield (Scheme 3) [53].

Other microorganisms also perform the oxidation of the exocyclic double bond. *R. stolonifer* was able to transform *ent*-kaurenoic acid (**1**) in the compound *ent-*16β,17-dihydroxy-kauran-19-oic acid (**42**) in a yield of 4%. The C-16 hydroxylation by the fungus *R. stolonifer*, as well as that performed by *G. fujikuroi*, occurred both in α-position (Scheme 3) [104].

**Scheme 4:**
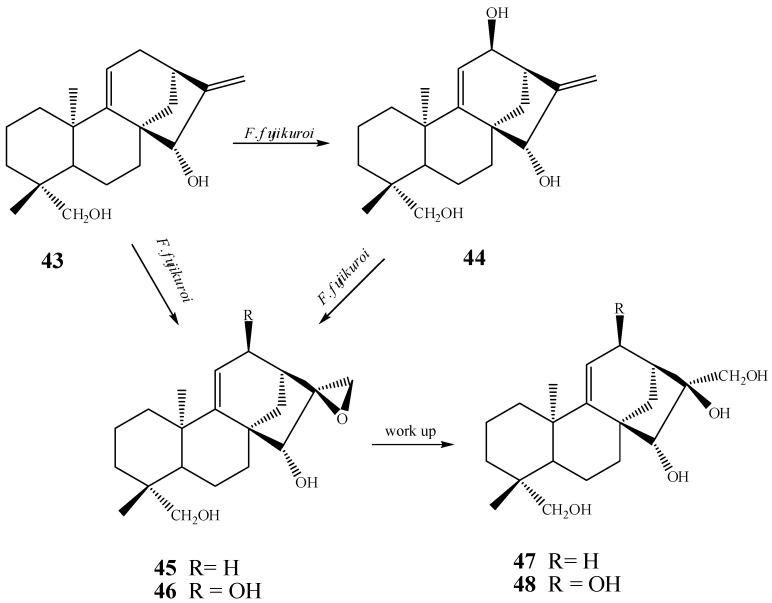
Route proposed by Fraga *et al.* [60] for obtaining compounds **47** and **48**.

Fraga *et al.* [60] showed a route involving compounds **43**–**46** in the biotransformation of kaurane derivatives with the fungus *F. fujikuroi*. The dihydroxylation of the positions 16 and 17 in *ent*-15β,16α,17,19-tetrahydroxy-kaur-9(11),16-diene (**47**) and *ent*-12α,15β,16α,17,19-pentahydroxy-kaur-9(11),16-diene (**48**) is derived from the formation of an epoxide with subsequent hydration of this group (Scheme 4). Fraga *et al.* proved that this last reaction is not performed by the fungus *F. fujikuroi*, but rather occurs naturally in the culture medium or during the purification procedure [60].

**Scheme 5:**
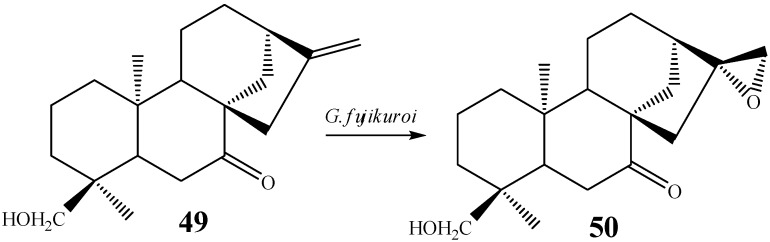
Epoxide formation by *G. fujikuroi.*

The proposal of a mechanism involving an epoxide at C-16/C-17 as intermediary in the biotransformation is corroborated by several other studies reporting epoxidation by fungi [62,89]. For instance, Fraga *et al.* [58] reported the action on *G. fujikuroi* on the substrate *ent*-7-oxo-18-hydroxy*-*kaur-16-ene (**49**) recovering an epoxide, *ent*-18-hydroxy-16β,17-epoxy-7-oxo*-*kaurane (**50**) (0.36% yield), in a stereoselective biotransformation (Scheme 5).

**Figure 12:**
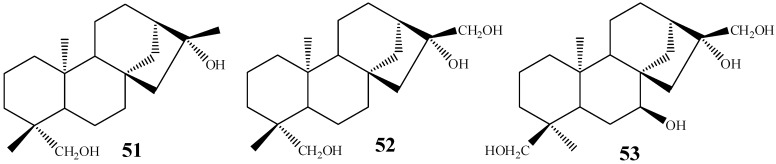
Chemical structures of compounds **51**–**53**.

The fungal action in the exocyclic double bond of kaurane diterpenes does not necessarily lead to diols. Rocha *et al.* [28] reported a good example of mono- and dihydroxylation of the 16-17 double bond by the same fungal species. The biotransformation of the compound *ent*-kaur-16-en-19-ol with *C. aphidicola* was able to generate two products: *ent-*kauran-16β,19-diol (**51**, 0.54%) and *ent-*kauran-16β,17,19-triol (**52**, 1.86%). In both compounds, the hydroxyl group inserted in position 16 has the same α-stereochemistry. Based on these results, it is possible that hydroxylation of C-17 ocurrs via the sequential hydroxylation of diol **51** [28]. Another C16-C17 diol bearing a 16α-hydroxyl group, *ent*-7α,16β,17,18-tetrahydroxy-kaurane **(53**), has been isolated from the biotransformation of epicandicandiol by *M. plumbeus* (Figure 12) [102].

**Scheme 6:**
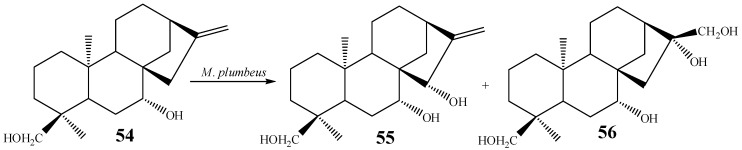
Biotransformation of candicandiol (**54**) into **55** and **56** by *M. plumbeus.*

Allylic hydroxylation at C-15 of kaurane diterpenes is another structural modification that has also been achieved by biotransformation. Introduction of a hydroxyl group at C-15 is one of the few reactions that also can be accomplished using chemical methods [106]. As an example, candicandiol (*ent*-7β,18-dihydroxy-kaur-16-ene, **54**) was incubated with the fungus *M. plumbeus*, being hydroxylated at C-15α to furnish canditriol (**55**) as a minor metabolite (1.47% yield) since a dihydroxylation product of (*ent*-7β,16β,17,18-tetrahydroxy*-*kaurane, **56**) was isolated in higher yield (15.08% yield) from the same experiment (Scheme 6) [102].

When comparing the yields of biotransformations performed with fungi of the genus *Mucor* [102] with other biotransformation experiments of kaurane diterpenes, it may be noticed that this species usually presents the best biotransformation yields. This genus is also reported to be efficient for the biotransformation of diterpenes with other skeletons [107,108].

## 6. Some Unconventional Biotransformations

**Figure 13:**
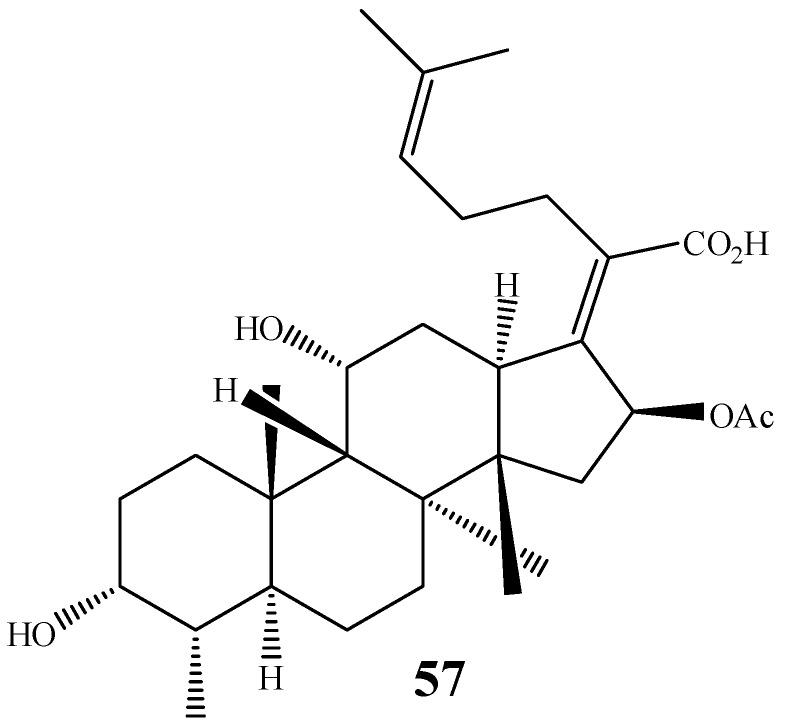
Chemical structure of fusidic acid (**57**).

The fungi have their own role in the hydroxylation of methylene groups. Comparing the literature available for fungal hydroxylation of, for instance, carbons 1, 11 and 12, all of them being methylene groups, C-1 and C-11 are spatially close to each other and subject to nearly the same spatial interactions, however, carbon 11 is much more frequently hydroxylated than carbon 1. One of the factors that may explain the preferential hydroxylation of carbon 11 may be related to the fact that this hydroxylation is a step of the biosynthesis of some triterpenes and steroids, such as fusidic acid (**57**) (Figure 13), commonly produced by fungus. These compounds have some structural similarities with the skeletons of diterpenes [109].

It has been reported that in the biotransformation of *ent-*kaur-16-ene derivatives by *G. fujikuroi*, a 15α- or 16α-hydroxyl group, or a 15α,16α-epoxide group inhibits oxidation at C-19 and directs hydroxylation to C-11β and C-7α [53,100].

**Figure 14:**
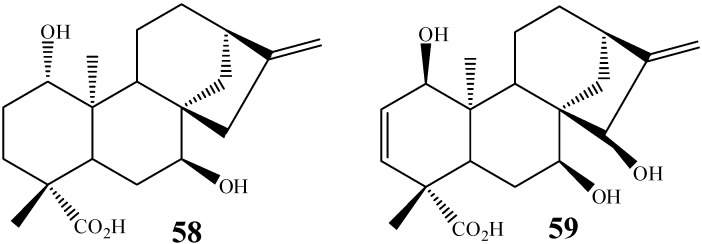
Chemical structures of kaurane diterpenes **58** and **59** bearing a C-1 hydroxyl group as a result of biotransformations.

*A. niger* is a fungal species known to introduce hydroxyl groups in kaurane diterpenes in positions C-7, C-11 [23,27], C-3 [65] and also at C-1, a non-conventional hydroxylation site. Kaurenoic acid, for instance, was incubated with *A. niger* to furnish the compound *ent-*1β,7α-dihydroxy-kaur-16-en-19-oic acid (**58**), bearing a hydroxyl group at C1 (5.8% yield) (Figure 14) [27]. Other works also report biotransformations of kaurane diterpenes resulting in products with C-1 hydroxylation by action of *A. blakesleeana* [64] and *G. fujikuroi* (compound **59**) [100].

An interesting comparison concerns the C-11 and C-12 positions. Observing a tridimensional model of the kaurane molecule at an appropriate angle, the latter seems to be much more available for an enzymatic hydroxylation. However, C-12 has not been reported to be a common site for biotransformation.

**Figure 15:**
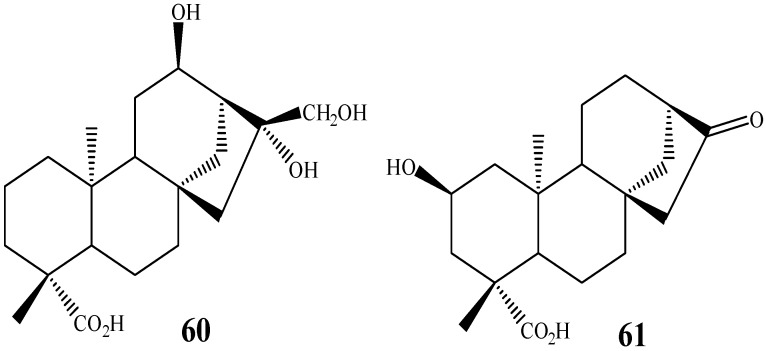
Chemical structures of kaurane diterpenes bearing C-12 and C-2 hydroxylations.

Pechwang *et al.* [103] reported the biotransformation of kaurenoic acid by *P. cubensis* (magic mushroom). Mushrooms have not been frequently used as biocatalysts in biotransformations. *P. cubensis*, used in this experiment, is reported to possess hallucinogenic effects and its metabolic profile comprises the psychotropic substances psilocybin and psilocin [110,111,112]. *P. cubensis* was able to hydroxylate kaurenoic acid at C-12 producing **60** (8.3% yield) (Figure 15) [103].

Hydroxylation of kaurane diterpenes at carbon 2 by biotransformation seems to have been first accomplished only in 2010 in the work of Rocha *et al.* [26]. In their work, *ent-*16-oxo-17-norkauran-19-oic acid, obtained from kaurenoic acid, was biotransformed by *F. proliferatum* producing the C-2 hydroxylated derivative *ent-*2α-hydroxy-16-oxo-17-norkauran-19-oic acid (**61**), with a yield of 17.6% (Figure 15).

**Scheme 7:**
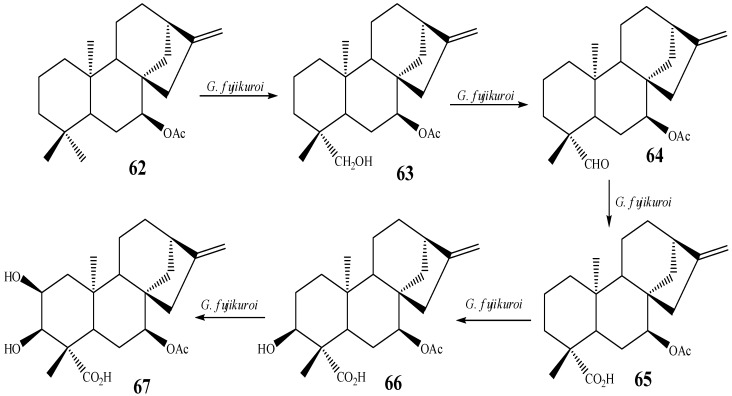
Route proposed by Fraga *et al.* for obtaining the compound *ent*-7α-acetoxy-2α,3α-dihydroxy-kaur-16-en-19-oic acid (**67**) by biotransformation using *G. fujikuroi*.

Later, Fraga *et al.* [10] reported that position C-2 could also be activated by the biotransformation of *ent*-7α-acetoxy-kaur-16-ene by *G. fujikuroi*. Fraga *et al.* proposed a biotransformation pathway from a sequence of oxidations that resulted in the formation of the product hydroxylated in C-2. The proposal is based on the products **62**–**67** obtained from their experiment and is shown in Scheme 7 [10].

Biotransformation resulting in C-15 allylic hydroxylation has been reported. The diterpene candicandiol, incubated with the fungus *M. plumbeus* produced the *ent*-7β,15β,18-trihydroxy-kaur-16-ene (**55**) [102].

## 7. Hydroxylations of Carbons 9 and 13

**Figure 16:**
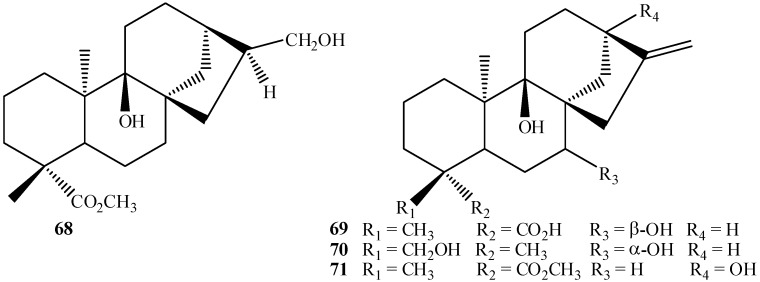
Chemical structures of some biotransformation products hydroxylated at C-9.

Although most common fungal hydroxylations in kaurane diterpenes occur at methylene carbons, the methine carbons C-9 and C-13 have also been activated by biotransformations. On the other hand, C-5 still seems not to have been the target of microbial hydroxylation so far. In relation to the methine carbon C-9, Vieira *et al.* [105] reported the hydroxylation of methyl *ent-*17-hydroxy-16βH-kauran-19-oate by *R. stolonifer*. The product *ent-*9α,17-dihydroxy-16S-kauran-19-oate (**68**) was isolated along with *ent-*7α,17-dihydroxy-16β-kauran-19-oate (12% conversion each) (Figure 16). In the biotransformation of kaurenoic acid by *A. blakesleeana* and by *R. oligosporus* the same C-9 hydroxylated product, *ent-*7α,9α-dihydroxy-kaur-16-en-19-oic acid (**69**), was obtained [64]. Hydroxylation at C-9 by biotransformation of candicandiol by *M. plumbeus* producing the C-9 hydroxylated compound *ent-*7β,9α,18-trihydroxy*-*kaur-16-ene (**70**) was also reported [102]. * R. stolonifer* is known to produce hydroxylation at position C-9 in steviol methyl ester to afford methyl *ent*-9α,13-dihydroxykaur-16-en-19-oate (**71**) [90].

**Figure 17:**
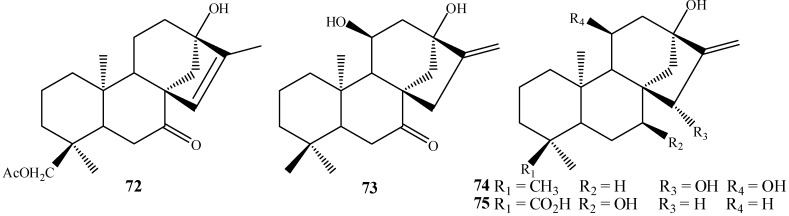
Products hydroxylated at positions C-11 and C-13.

The fungus *R. nigricans* was able to insert a C-13 hydroxyl group in the compound *ent-*18-acetoxykaur-15-en-7-one in a regioselective biotransformation to furnish the compound *ent-*18-acetoxy-13-hydroxykaur-15-en-7-one (**72**), in 27.83% yield [62]. However, it seems to be more common to observe hydroxylation of C-13 accompanied by hydroxylation of other carbons. For instance, the compound *ent*-7-oxo-kaur-16-ene, when incubated with *G. fujikuroi*, furnished the diol *ent*-11α,13-dihydroxy-7-oxo-kaur-16-ene (**73**) [58]. Fraga *et al.* [92] isolated another biotransformation product **74** with C-11 and C-13 hydroxylation produced by the fungus *G. fujikuroi*. In biotransformation of kaurenoic acid by *A. blakesleeana* hydroxylation of C-13 was accompanied by introduction of a 7α-OH into the product, to give *ent-*7β,13-dihydroxy-kaur-16-en-19-oic acid (**75**) (Figure 17) [64].

## 8. Hydroxylation of Kaurane Diterpene Methyl Groups

Kaurane diterpenes often possesses C-18 and C-20 and eventually C-17 as non-functionalized methyl groups [113,114] although is more common to find a double bond between C16 and C-17. Position 19 may be oxidized as a carboxyl group or a primary alcohol. Considering the four possible methyl groups on the carbon skeleton, the methyl groups C-19 and C-20 are the more sterically hindered because they experience 1,3-diaxial interactions between each other. The C-18 methyl group is in an equatorial position, and therefore, in theory, it should be more available to enzymatic attack.

In relation to the presence of a C-17 methyl group attached to C-16, in theory, a 16α-CH_3_ should be more exposed. In the α-position, this methyl group suffers less hindrance than a 16β-CH_3_ since the latter is close to the C-11 methylene group, considering ring C in the preferential chair conformation.

**Scheme 8:**
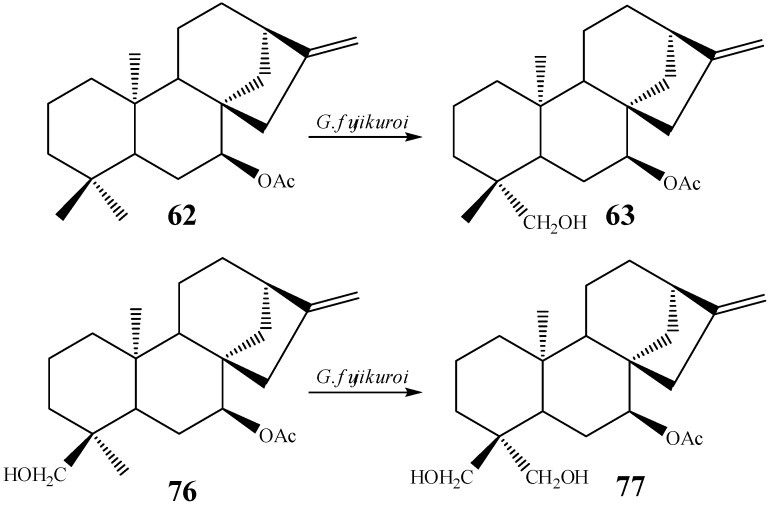
Hydroxylation of methyl C-19 by *G. fujikuroi.*

However, microbial hydroxylation relies more on the microorganism’s features and most of them can overcome steric hindrance. For instance, hydroxylation at C-19 was achieved by incubating *ent-*7α-acetoxy-kaur-16-ene (**62**) with *G. fujikuroi* to furnish *ent-*7α-acetoxy-19-hydroxy-kaur-16-ene (**63**) (1.26% yield) [10]. When a kaurane derivative hydroxylated at the C-18 methyl group (*ent-*7α-acetoxy-18-hydroxy-kaur-16-ene, **76**) was used as substrate in a biotransformations experiment using the same fungus, hydroxylation at C-19 was also achieved, leading to the formation of *ent-*7α-acetoxy-18,19-dihydroxy-kaur-16-ene (**77**), in 2.14% yield (Scheme 8) [10].

In the studies with *G. fujikuroi*, the derivative containing a hydroxyl group at position 19 does not suffer a subsequent oxidation on carbon 18. Following the possible biosynthetic routes used by this species, it was noticed that after the hydroxylation of C-19, several oxidations occur, including hydroxylation at C-3 prior to hydroxylation at position 18 [10].

**Scheme 9:**
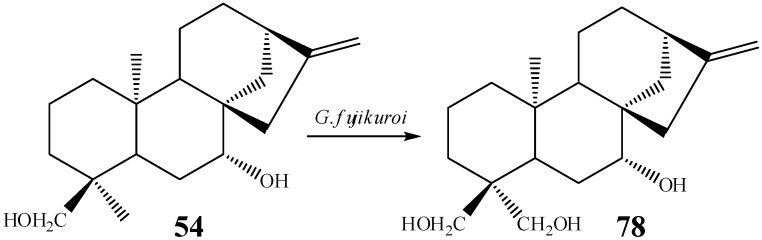
Biotransformation of candicandiol by *G. fujikuroi.*

The incubation of candicandiol (**54**) with the fungus *G. fujikuroi* led to the isolation of a derivative hydroxyated at C-19 (*ent*-7β,18,19-trihydroxy-kaur-16-ene, **78**), in 4.5% yield (Scheme 9) [53]. When the substrate used in this biotransformation contained an acetyl group at C-7, the yield of the C-19 hydroxylation was lower [10].

**Scheme 10:**
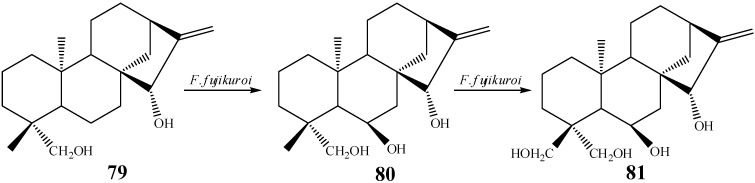
Route proposed by Fraga *et al.* for the hydroxylation of carbon 18.

In another work, a substrate not functionalized at C-7 was used, and in this case it was possible to obtain the hydroxylation of methyl 18 when carbon 19 was hydroxylated. The authors proposed that the biotransformation of **79** by *F. fujikuroi* started with the hydroxylation at position C-6β, based on the isolation of compound *ent*-6α,15β,19-trihydroxy-kaur-9(11),16-diene (**80**) from this experiment, followed by hydroxylation at C-18 to furnish the product *ent-*6α,15β,18,19-tetrahydroxy-kaur-9(11),16-diene (**81**, Scheme 10). Among the derivatives isolated from this biotransformation, the compound hydroxylated at C-19 was produced in lower yield and presented lower stability [60].

**Scheme 11:**
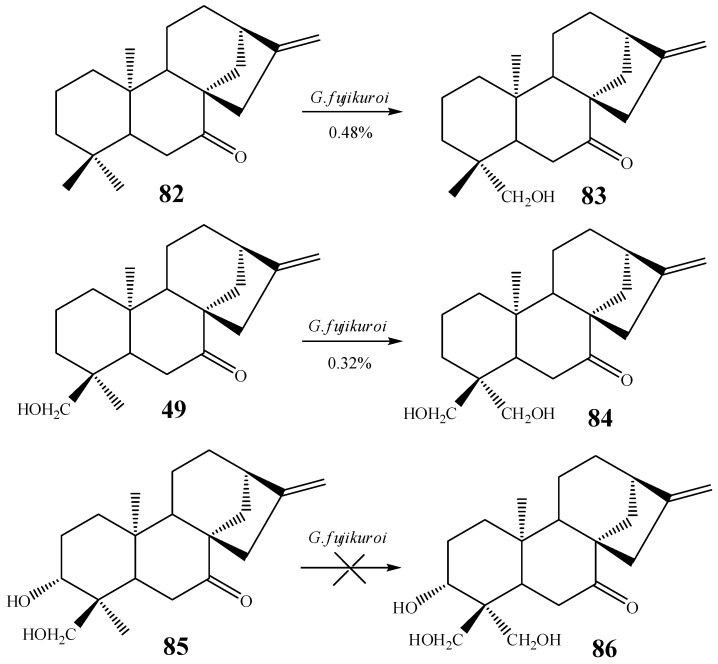
C-19 biotransformation using different products by *G. fujikuroi.*

In an attempt to study the correlation between hydroxylation at C-18 and C-19 positions, Fraga *et al.* carried out a study using the compound *ent*-7-oxo-kaur-16-ene and derivatives containing different substitution patterns at C-3 and C-18 (compounds **82**, **49**, **85**, Scheme 11). The compounds were incubated with the fungus *G. fujikuroi* [58].

Compounds **83** and **84** were hydroxylated at C-19 while compound **86**, a derivative hydroxylated at C-3, was not hydroxylated at C-19 by *G. fujikuroi* [58].

## 9. Structural Alterations Other than Hydroxylation

Hydroxylations are the structural modifications most often reported as a result of biotransformations of kaurane diterpenes using fungi, but the capacity of such microorganisms to modify the structure of this class of natural products is even greater.

**Scheme 12:**
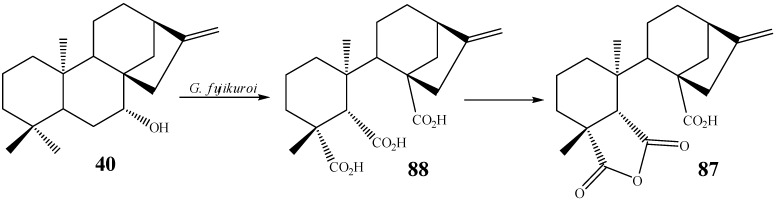
Ring opening reaction by *G. fujikuroi.*

Other modifications reported include ring rearrangements with formation of gibberellane [53,56,57,115] and beyerane diterpenes [23,43,116], formation of lactones [21,58,60], epoxidation [60], glycosylation [21], alkene hydrogenation [100], ring opening [53], decarboxylation [60] and the formation of alkenes [43]. Oxidations and ring opening were reported by Fraga *et al.* When *epi*-candol A (**40**) was submitted to biotransformation by the fungus *G. fujikuroi,* two products were obtained, *ent-*7β,16β,17-trihydroxy-kaur-16-ene and fujenoic acid (**87**) [53]. Fujenoic acid was formed after hydroxylation at C-6, followed by oxidation at C-7 and ring opening (Scheme 12). The *fujenoic* triacid **88** is a natural product found in *G. fujikuroi* and it is an intermediate of gibberellin biosynthesis [117]. To ensure that the product obtained in this biotransformation was not a secondary metabolite of this fungus, the experiment was repeated with isotopic labeling of the substrate [53].

**Scheme 13:**
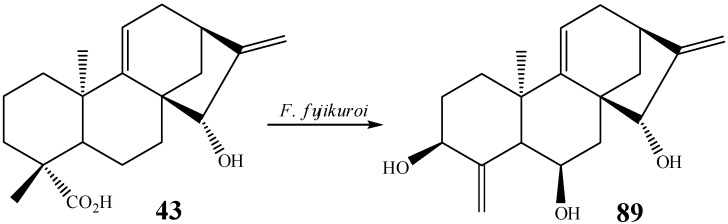
Formation of exocyclic double bond at C-4 by *F. fujikuroi.*

Another interesting modification reported is the decarboxylation of *ent*-15β-hydroxy-kaur-9(11),16-dien-19-oic acid (**43**) with the fungus *F. fujikuroi.* Products bearing new hydroxylations at C3β and C-6β and 19,6α-lactone derivatives were obtained, besides an oxidative decarboxylation product affording a compound **89** bearing a 4,18-double bond (Scheme 13). A mechanism for the formation of this exocyclic double bond after decarboxylation of the carboxyl group has been proposed by Fraga *et al.* [60].

**Scheme 14:**
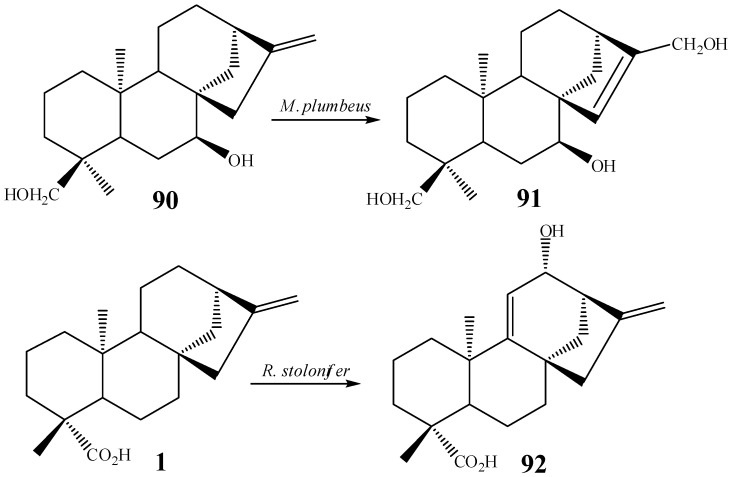
Double bond formation by *M. plumbeus* and *R. stolonifer.*

Isomerization of the exocyclic double bond in **90** of the starting material epicandicandiol (**91**) has been reported utilizing the fungus *M. plumbeus* [102]. The formation of a double bond was also observed in the biotransformation by the fungus *R. stolonifer* in another position of the kaurane skeleton (between carbons 9 and 11, *i.e.*, compounds **1**, **92**, Scheme 14) [104].

**Figure 18:**
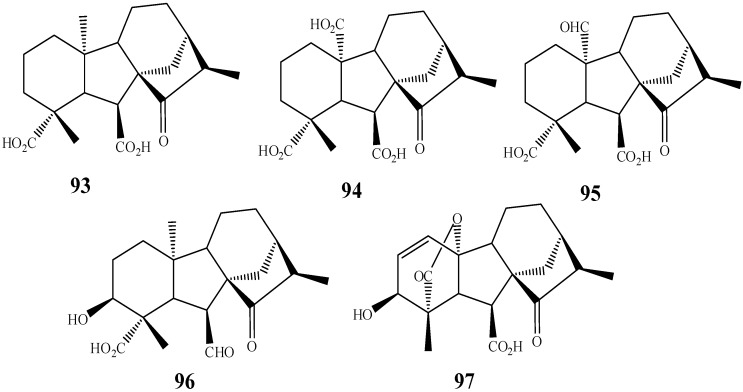
Rearrangement product formed in the biotransformation of 7-oxo-*ent*-kaur-16-ene by *G. fujikuroi.*

In addition to the already discussed changes that can occur in the kaurane skeleton, rearrangements leading to other classes of diterpenes have been reported, being interesting as a methodology to synthesize compounds with other skeletons. For instance, the diterpene *ent*-15-oxo-kaur-16-ene furnished seven biotransformation products, five of them (**93**–**97**, Figure 18) belonging to the gibberellin group by the action of the fungus *G. fujikuroi* [57]. 

**Figure 19:**
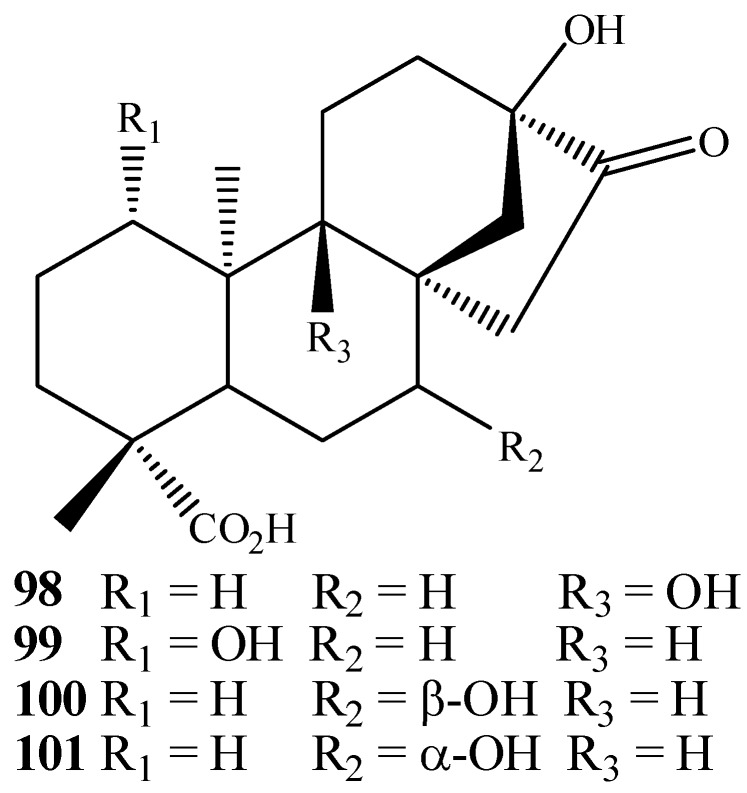
Chemical structures of rearrangements products obtained from biotransformation by *C. bainieri.*

Another interesting rearrangement of kaurane diterpenes can be exemplified by the biotransformation of steviol-16α,17-epoxide by *C. bainieri* that furnished *ent*-9α,17-dihydroxy-16-ketobeyeran-19-oic acid (**98**), *ent*-1β,17-dihydroxy-16-ketobeyeran-19-oic acid (**99**), *ent*-7α,17-dihydroxy-16-ketobeyeran-19-oic acid (**100**) and *ent*-7β,17-dihydroxy-16-ketobeyeran-19-oic acid (**101**) as rearrangement products displying beyerane skeletons (Figure 19) [43].

## 10. Final Remarks

In this review we have showed selected examples of the structural diversity of kaurane diterpenes obtained as a result of biotransformations by filamentous fungi species. Kaurane skeletons have been functionalized on all four rings and in both, α- and β-stereochemistry at most of the carbons. Such functionalizations of molecules represent an important strategy in the discovery of new drugs with different activities.

Advances in the field of natural products research already allow working with intelligent design of novel pharmacologically active molecules. Pharmaceutical chemistry is one of the fields that deal with the creation of molecules for specific goals. The success of this approach is highly dependent on a complete understanding of substrate skeletal susceptibility to chemical and microbial funcionalizations. The intersection of information about structural features responsible for biological activities and fungal species able to activate the structures in these required positions can be extremely useful for the intelligent design of new kaurane derivatives with enhanced biological activities.

Biotransformation of terpenes is an area that is expected to grow since it remains as a modern alternative in the gap left by organic synthesis, due to the possibility of activating methylene carbons. The scope of biotransformations and the green features associated to this methodology are also expected to be a lever feature for increasing the scope of biotransformations used in the new drug discovery process.
